# A systematic review and meta-analysis on the clinical implications of probability discounting among individuals with Internet gaming disorder

**DOI:** 10.1038/s41598-021-82822-z

**Published:** 2021-02-04

**Authors:** Weilun Chung, Cheuk-Kwan Sun, I.-Ting Tsai, Kuo-Chuan Hung, Hsien-Jane Chiu, Ruu-Fen Tzang, Pin-Yang Yeh, Yu-Shian Cheng

**Affiliations:** 1 Department of Psychiatry, Tsyr-Huey Mental Hospital, Kaohsiung Jen-Ai’s Home, No. 509, Fengping 1st Rd., Houzhuang Vil., Daliao Dist., Kaohsiung City, 831 Taiwan; 2 Department of Emergency Medicine, E-Da Hospital, Kaohsiung, Taiwan; 3 School of Medicine, College of Medicine, I-Shou University, Kaohsiung, Taiwan; 4 Department of Anesthesiology, Chi Mei Medical Center, Tainan, Taiwan; 5 Department of Health and Nutrition, Chia Nan University of Pharmacy and Science, Tainan, Taiwan; 6 Taoyuan Psychiatric Center, Ministry of Health and Welfare, Taoyuan City, Taiwan; 7 Institute of Hospital and Health Care Administration, National Yang-Ming University, Taipei City, Taiwan; 8 Department of Psychiatry, Mackay Memorial Hospital, Taipei, Taiwan; 9 Department of Psychology, College of Medical and Health Science, Asia University, No. 500, Lioufeng Rd., Wufeng, Taichung, 41354 Taiwan; 10 Institute of Biomedical Sciences, National Sun Yat-Sen University, Kaohsiung City, Taiwan

**Keywords:** Psychology, Health care, Neurology

## Abstract

The significance of probability discounting (PD) among individuals with Internet gaming disorder (IGD) remains unclear. Following the PRISMA guidelines, we systematically searched the PubMed, Embase, and ScienceDirect databases for English articles on Internet addiction that included comparison between individuals with and without IGD as well as probabilistic discounting task as the main outcome from January 1970 to July 2020 using the appropriate keyword strings. The primary outcome was the overall difference in rate of PD, while the secondary outcomes included the difference in PD with magnitude of probabilistic reward and response time of the PD task. Effect size (ES) was calculated through dividing the group means (e.g., *h* value or AUC) by the pooled standard deviations of the two groups. A total of five studies with 300 participants (i.e., IGD group, n = 150, mean age = 20.27 ± 2.68; healthy controls, n = 150, mean age = 20.70 ± 2.81) were analyzed. The IGD group was more willing to take risks in probabilistic gains but performances on probabilistic losses were similar between the two groups. The IGD group also exhibited a shorter response time (Hedge’s *g* = − 0.51; 95%CI = − 0.87 to − 0.15). Meta-regression demonstrated a positive correlation between maximum reward magnitude and PD rate (*p* < 0.04). However, significant publication bias was noted among the included studies (Egger’s test, *p* < 0.01). In conclusion, individuals with IGD seemed more impulsive in making risky decisions, especially when the potential gains were expected. Our findings not only supported the use of PD for assessing individuals with IGD but may also provide new insights into appropriate interventions.

## Introduction

Internet gaming disorder (IGD) is increasingly being considered to be a distinct clinical entity among all the documented addictive disorders. The American Psychiatric Association (APA) classified IGD as a tentative disorder in need of further study in the latest (fifth) edition of the Diagnostic and Statistical Manual of Mental Disorders (DSM-5)^1^ and the World Health Organization (WHO) recognized it as an official diagnostic entity in the latest (eleventh) revision of the International Classification of Diseases (ICD-11)^2^. According to the IGD DSM-5 criteria, those suffering from IGD not only spent excessive time in gaming, but also experiencing symptoms similar to addictive behaviors, such as “the need to spend more time gaming to satisfy the urge” and withdrawal symptoms when gaming is taken away. Therefore, the addictive criteria are essential to the diagnosis of IGD. The reported prevalence of IGD varies widely, ranging from 1% in selected samples^3^ to as high as 57.5% in general populations^4^ due to the differences in diagnostic approaches. Children and adolescents are particularly susceptible because of age-related underdevelopment of cognitive control^5^. Instead of being an isolated occurrence, the disorder is frequently associated with other psychiatric conditions^4^, especially attention-deficit hyperactivity disorder (ADHD) in which the altered executive control networks may predispose to the development of IGD^6^.

Although the mechanism underlying IGD remained unclear, studies have shown that participants with IGD may make more impulsive decision compared with healthy control^6^. Neuroimaging studies also demonstrated that participants with IGD showed decreased frontal brain responses during processing of losing outcomes, suggesting their decreased sensitivity to losses during decision-making. The finding may explain their poor impulse control similar to that in those suffering from substance use disorders^6,7^. Impulsive choice is often regarded as risk-based decision-making, while delay discounting (DD) and probabilistic discounting (PD) both are important methods for investigating the underlying behavioral mechanisms^8^. During PD performance, participants made a series of choices between large, uncertain rewards and small, certain rewards^9^. In other words, PD is a method for investigating the relation between the subjective value of a reward and the likelihood of its receipt. Therefore, PD is actively being applied to the study of behaviors (e.g., food intake^10^, smoking^11^, alcohol consumption^12^, sexuality^13^, and gaming^14^) as well as in certain clinical groups (e.g., ADHD^15^, pathological gambler^16^, obesity^17^, and substance user^18^) and populations (e.g., adolescents^19^).

**Figure 1:**
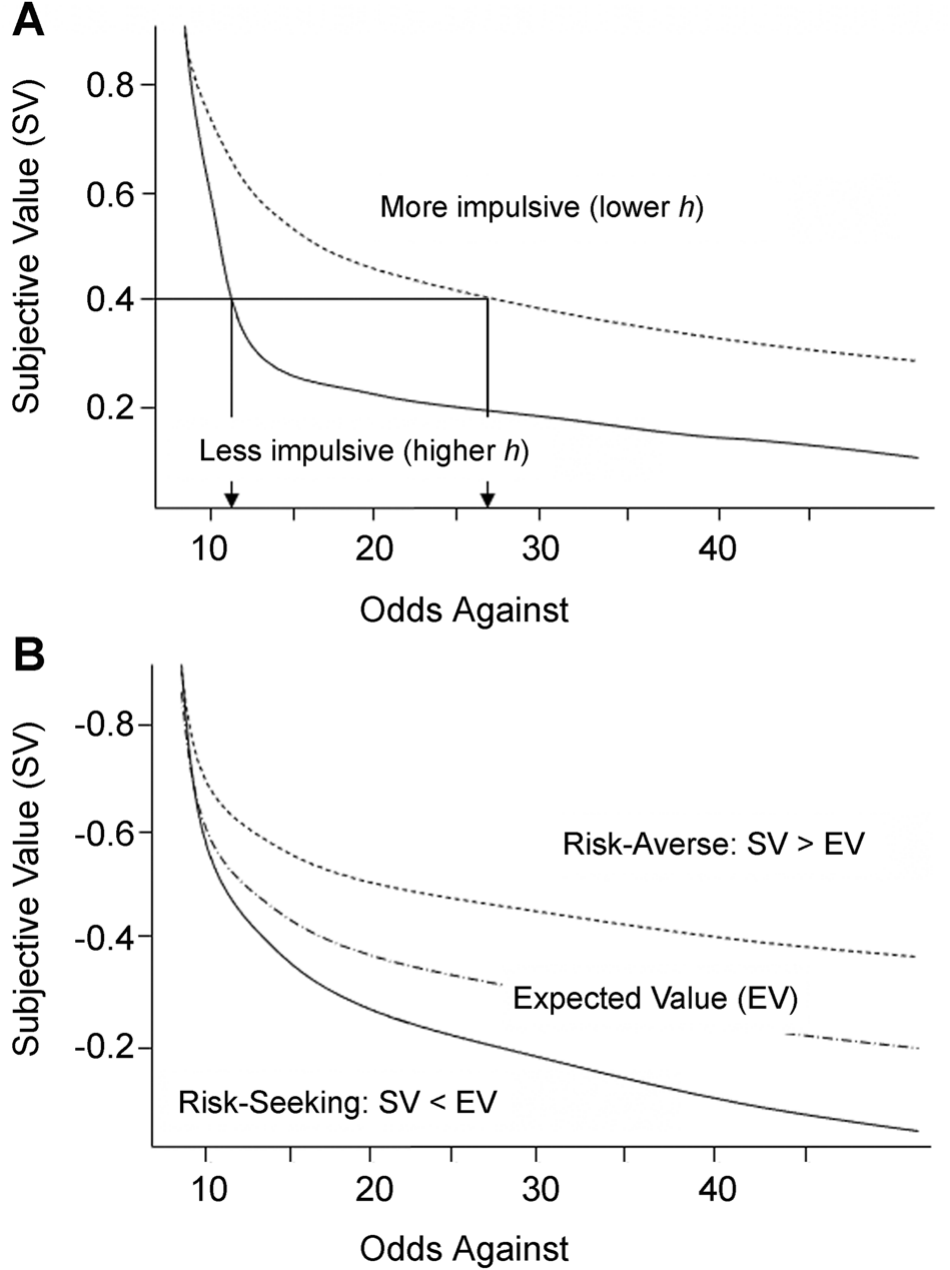
Probabilistic discounting functions. (**A**) Probability discounting of gains, and (**B**) Probability discounting of losses.

When compared with normal controls, risk-seeking individuals were found to show shallower discounting curves of probabilistic gains due to their overestimating the possibility of a substantial gain and underestimating the likelihood of gaining nothing^9^ (Fig. 1). Figure 1A illustrates the difference in the estimation of odds against (i.e., the ratio of the number of unfavorable outcomes to that of favorable outcome) between individuals with low and high impulsivity [i.e., higher and lower rate of PD (*h*), respectively] with the same subjective reward value. On the other hand, Fig. 1B shows that the discounting curves of individuals with risk-seeking behavior were steep as they overvalue the possibility of losing nothing and undervalue the possible outcome of a great loss^20^. The hyperbolic model of probability discounting functions^21,22^ has two free parameters: the parameter *h*, which reflects the discounting rate (i.e., the value of the probabilistic gain is discounted), and the parameter *s*, which governs the shape of the discounting function^22^.

It is important to understand the behavioral mechanism underlying IGD as it may not only help in making correct diagnosis but also guide behavioral interventions. PD serves as an important behavioral measure to understand impulsive choices in different types of addictions, also in individuals with IGD^23,24^. However, to date, the significance of PD in individuals with IGD has not been systemically investigated. For instance, it remains unclear whether PD can serve as an assessment parameter for those with IGD. Furthermore, the issues of a possible association between the rate of PD and the amount of probabilistic gain (i.e., the amount effect) as well as the potential asymmetry in the rate of PD between probabilistic gains and losses among subjects with IGD have not been adequately addressed. Therefore, the present meta-analysis aimed at exploring the therapeutic implications of PD in the IGD population through reviewing currently available clinical evidence.

## Methods

### Study eligibility and definitions

**Figure 2:**
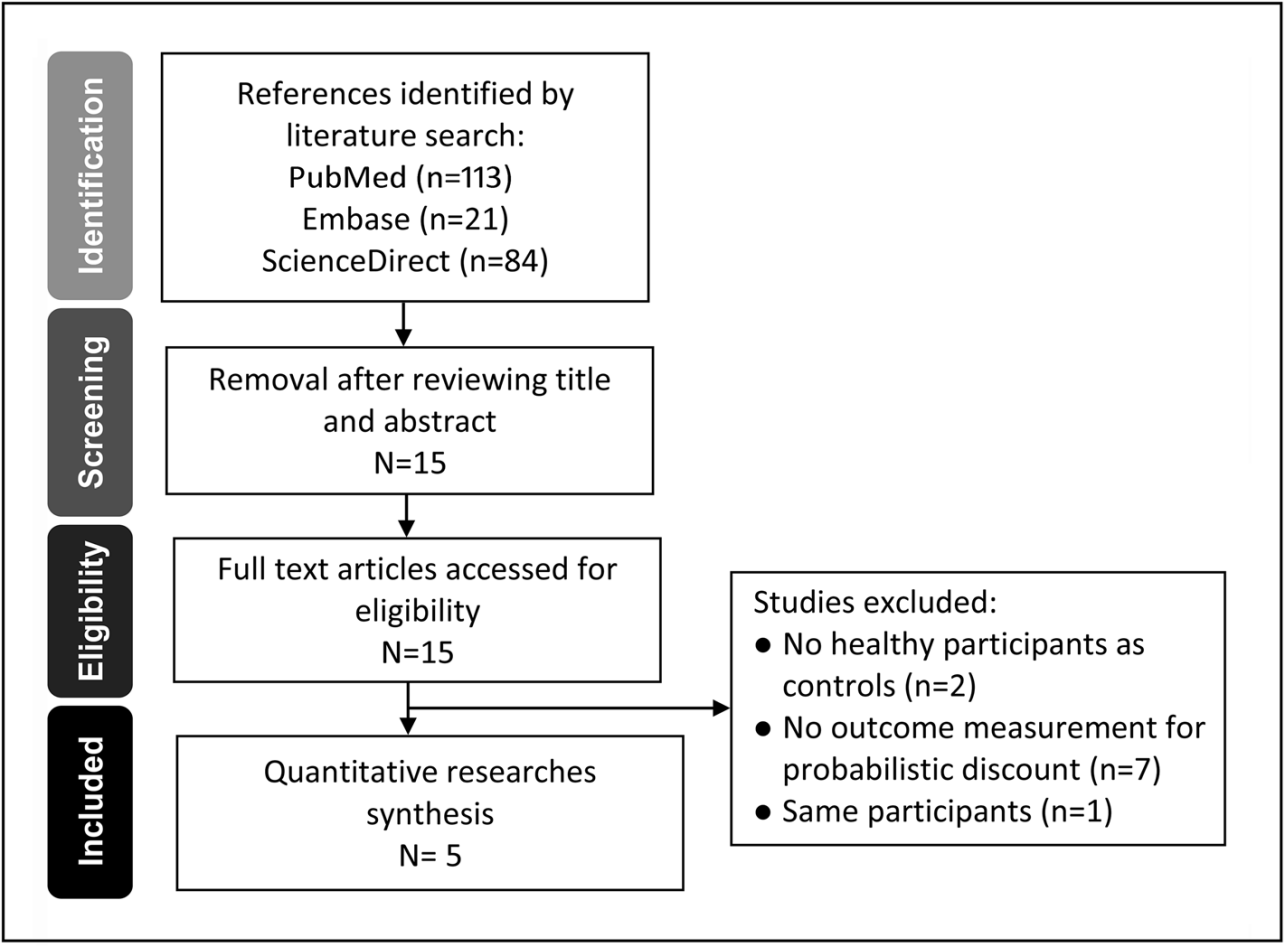
PRISMA flowchart for identifying eligible studies.

Although we aimed at investigating IGD, keywords describing Internet addiction were also used in a string to search for the eligible articles. Critical terms across different fields that included psychological, psychiatric, and neuroscientific literature have been used to refer to an addiction to the Internet^25^. Different keyword strings were used to ensure the completeness of literature search, namely, *Internet addiction* OR *problematic Internet use* OR *pathological Internet use* OR *excessive Internet use* OR *Internet dependence* OR *compulsive Internet use* OR *compulsive computer use* OR *virtual addiction* OR *Internet use* OR *pathologic use of Internet* OR *Internet behavioral addiction* OR *Internet abuse* OR *Internet overuse* OR *harmful use of the Internet* OR *Internet addictive disorder* OR *Internet gaming disorder* AND *probabilistic reward discounting* OR *probability discounting* OR *impulsive choice*. The inclusion criteria for studies were as follows: (1) those focusing on using the Internet for gaming, but not for accessing information, cybersex, gambling or social network, (2) those in which the severity of IGD could be assessed or diagnosed, (3) those involving a comparison between individuals with IGD and those without, and (4) those employing probability discounting task as the main outcome. In contrast, studies on medications (e.g., antidepressants, antipsychotics), severe mental illness (e.g., mood and psychotic disorders), and/or neurological diseases (e.g., dementia, Parkinson’s disease) were excluded. Figure 2 is a flowchart of the present investigation, depicting the process of identifying eligible studies.

### Electronic searches

According to the Preferred Reporting Items for Systematic Review and Meta-Analysis (PRISMA)^26,27^ and the Meta-analysis Of Observational Studies in Epidemiology (MOOSE) statement^28^, we systematically searched the PubMed, Embase, and ScienceDirect databases for eligible English articles from inception to July 2020.

### Study selection

Two corresponding authors (Yeh PY and Cheng YS) completed the title and abstract screening. The full-text screening stage was also independently carried out by both authors (Yeh PY and Cheng YS). Yeh PY considered six studies to be eligible but Cheng YS only included five, giving an inter-observer reliability of 83.3% (i.e., disagreement rate of one in six). A third author (Sun CK) was consulted and decided to exclude that study because of a potential overlapping of subjects in one study^14^ with those of another^29^.

### Data synthesis

The primary outcome was the overall change in rate of PD, while the secondary outcomes included the change in PD with the amount of probabilistic reward (i.e., the amount effect), response time (RT) of the PD task as well as the difference in PD between probabilistic gains and losses. Effect size (ES) was calculated by group means [e.g., *h* value or area under the curve (AUC)], and dividing the result by the pooled standard deviations of the two groups. In addition to the *h* value from the equation for hyperbolic PD function as previously mentioned, AUC is also used to analyze PD^30^. AUC, instead of *h* value, is often adopted to measure PD because of its characteristics (i.e., unskewed distribution and no mathematical assumptions)^30^. We used the computer program “Comprehensive Meta-Analysis version for Windows (CMA, version 2.2.064)” to calculate pooled mean ESs. ESs indicating the performance on PD tasks were calculated by using a negative sign for *h* value but a reversed one for AUC. A smaller ES in PD indicates that individuals with IGD were more prone to making impulsive decisions compared with healthy controls. When a study used more than one validated measure to represent the PD, the results of multiple measures classified in the same domain were standardized and averaged to produce a single ES. ESs of 0.8 are interpreted as large, while ESs of 0.5 are medium, and ESs of 0.2 are small^31^. Since a small sample size can decrease statistical power, this meta-analysis adjusted for small sample bias by using Hedges’ *g* rather than Cohen’s *d* as previously suggested^32^*.* Besides, we used the random-effects model because of similar weights across studies^33^ and decreased heterogeneity^34^. Moreover, a sensitivity analysis was conducted using the leave-one-out method (i.e., removing one study each time and repeating the analysis) to evaluate the influence of each study on the overall ES^35^.

Subgroup analyses were also conducted according to a random-effects model^32^, and we calculated *Q* statistics as indicators of heterogeneity. Additionally, we used mixed-effects meta-regression analyses for the moderators (e.g., age, the percentage of female, and task-related variables) to test for a significant relationship between the continuous variable and ES. Publication bias was tested by inspecting the funnel plot on the outcome measures of PD tasks and by adopting Duval and Tweedie’s trim and fill procedure^36^, which yields an estimate of the ES after adjusting for publication bias. When there was evidence of funnel plot asymmetry, potentially missing studies were imputed using the ‘trim and fill’ method^36^. Finally, we performed Egger’s test to assess whether the bias captured by the funnel plot was significant.

## Results

### Study characteristics

Because two studies^14,29^ shared the same subjects, we only selected one^29^ for the present study. Therefore, although six studies were identified, the data from five studies were included in the current meta-analysis. There were 150 individuals with IGD (mean age = 20.27 years, SD = 2.68 years) and 150 healthy controls (mean age = 20.70 years, SD = 2.81 years), giving a total of 300 participants for the present study. The characteristics of the included studies are shown in Table 1. Of the five included studies, only two included both genders^37,38^, while the other three included only males. Of the five studies, four used the DSM-5 diagnosis of IGD, but one study by Li et al.^37^ adopted Young’s diagnostic questionnaire which is an evaluation tool for Internet addiction. Nevertheless, participants in that study spent more than 50% of their online time playing games.

**Table 1:** Comparison between subjects with Internet gaming addiction and healthy controls in probability discounting task.

Studies	IGD (N); CON (N)	Age	Edu (years)	Female (%)	Severity of IGD	Gaming duration (h/day)	Consecutive choices for each of probabilities	Probability options (%)	RT (ms); blank (ms); magnitude of first adjustment	Max. reward (USD)	Analysis	Country
Li et al.^37^	28; 28	21.09	15.86	58.90	6.07 (YDQ)	3.73	7	95, 75, 55, 25, 10, 5	NA; NA; half of the difference between guaranteed and risky choices	3654	AUC	China
Tian et al.^38^	42; 41	15.67	9.76	48.20	DSM-5	4.62	7	95, 75, 50, 30, 10, 5	NA; NA; one fourth	7307	AUC	China
Wang et al.^29^	42; 41	22.52	> 13	0.00	DSM-5	n/a	9	90, 80, 70, 60, 50, 40, 30, 20, 10	400; 500; NA	15	*h* value	China
Wang et al.^39^	18;20	21.45	14.52	0.00	DSM-5	n/a	9	90, 80, 70, 60, 50, 40, 30, 20, 10	400; 500; NA	15	*h* value	China
Wang et al.^40^	20; 20	21.73	> 13	0.00	DSM-5	2.89	9	90, 80, 70, 60, 50, 40, 30, 20, 10	400; 500; NA	15	*h* value	China

AUC, area under the curve; CON, control group; DSM-5, Diagnostic and Statistical Manual of Mental Disorders, 5th Edition; Edu, duration of education from primary school onward; IGD, Internet gaming disorder; N, number of subjects; NA, not available; RT, reaction Time; YDQ, Young’s diagnostic questionnaire for Internet addiction; YIAT, Young’s Internet addiction test.

The options of probabilistic values across the five included studies were slightly different; three used nine consecutive probability values, namely 10%, 20%, 30%, 40%, 50%, 60%, 70%, 80%, 90%^29,39,40^, one employed PD task composing of seven probabilities (i.e., 95%, 75%, 50%, 30%, 10%, and 5%)^38^, and one utilized probabilistic decision-making consisting of six different risk levels (i.e., 95%, 75%, 55%, 25%, 10%, and 5%)^37^. Besides, the maximum magnitude of monetary reward varied widely from 100 Yuan (14 USD)^29,39,40^ to 50,000 Yuan (over 7000 USD)^38^. The *h* value and AUC indicated the performance on PD task as PD rate. Besides, all studied subjects were ethnically Chinese.

### Quantitative data synthesis

**Figure 3:**
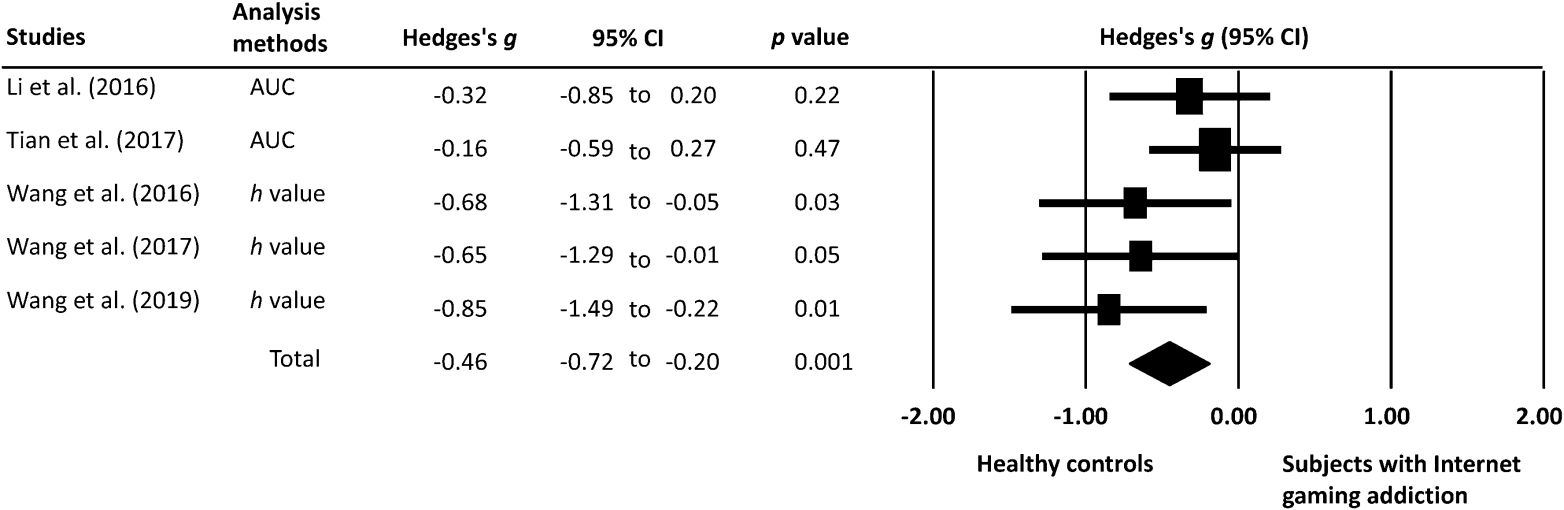
Forest plot of the effect sizes for the difference in probability discounting between subjects with Internet gaming disorder and healthy controls. The bars with squares in the middle denote 95% confidence intervals (95% CIs) and effect sizes (ESs). The diamond represents the pooled effect size (ES).

**Figure 4:**
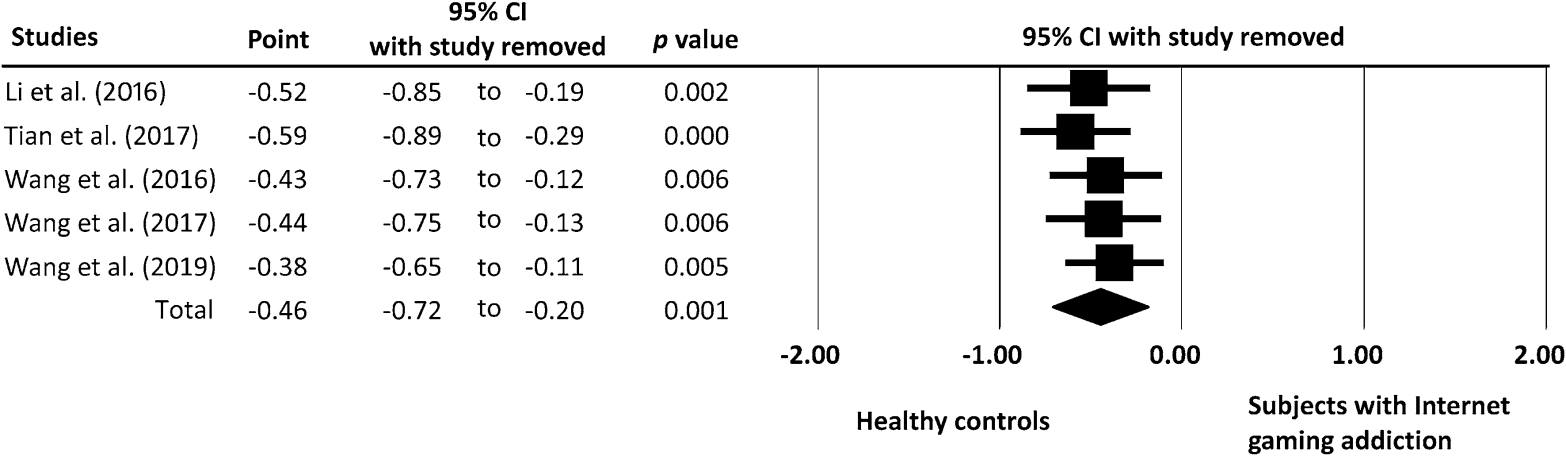
Leave-one-out sensitivity analysis. The bars with squares in the middle denote 95% confidence intervals (95% CIs) and effect sizes (ESs). The diamond represents the pooled effect size (ES).

**Figure 5:**
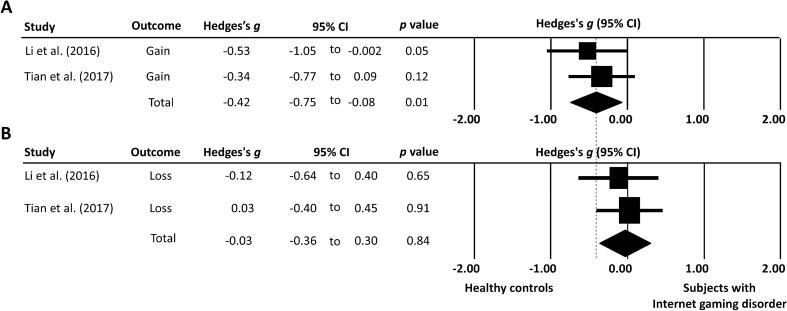
Forest plot of the effect sizes for the difference in probabilistic (**A**) gain, and (**B**) loss between subjects with Internet gaming disorder and healthy controls. The bars with squares in the middle denote 95% confidence intervals (95% CIs) and effect sizes (ESs). The vertical dotted line indicates the ESs for null hypothesis. The diamond represents the pooled effect size (ES).

This meta-analysis of data from five studies found a significant difference in PD rate between individuals with IGD and those without (Fig. 3). The ES was strong in the leave-one-out sensitivity analysis (Fig. 4), suggesting that the main result was not driven by any single study. Figure 5 showed that individuals with IGD were more willing to take risks involved in probabilistic gains than their healthy counterparts. However, their performances on probabilistic losses were similar. Besides, of the five included studies, three^29,39,40^ provided RT of the probability discounting task. The ES for RT (Hedges’s *g* = − 0.51; 95% CI = − 0.87 to − 0.15) was significant (*p* = 0.006), indicating that individuals with IGD spent less time on making risky choices.

As for data analyzed by different methods, the participants with IGD demonstrated a significantly lower PD rate than that of healthy control when *h* value was used, but showed only borderline significance when using the AUC method (Table 2). Although statistical analysis revealed significant heterogeneity in the methods for analyzing PD rate (*p* < 0.05), the difference in ES between the two methods was not significant (Table 2).

**Table 2:** Comparison of the significance of difference in assessing the rate of probability discounting between two analytical methods.

Method	Number of studies	*g*^*a*^	95% CI	Z	*Q*^b^	*P*^c^
AUC	2	− 0.23	− 0.56 to 0.11	− 1.33	3.96^#^	0.06
*h* value	3	− 0.73	− 1.09 to − 0.36	− 3.89		

AUC, area under the curve.

^a^According to the random effects model.

^b^Cochran’s *Q* for heterogeneity assessment in accordance with random effects analysis.

^c^Significance of difference between the effect sizes in subgroups; ^#^*p* < 0.05.

### Meta-regression

Mixed-effects meta-regression was performed to evaluate the effect of moderators (Table 3). The regression coefficients for the percentage of female had a trend toward statistical significance in response to an increase in ES (*p* = 0.057). Furthermore, the maximum magnitude of reward positively correlated with the PD rate (*p* < 0.04), suggesting a positive association between the maximum magnitude of reward and the PD rate. However, other mediators showed no significant impact on the PD rate (all *p* > 0.08).

**Table 3:** Regression coefficients using the mixed effect model for studies on probability discounting task.

Variable (continuous)	Coefficient (95% CI)	*P*
The percentage of female	0.009 (− 0.0003 to 0.018)	0.057
Age	− 0.07 (− 0.16 to 0.01)	0.08
Education	− 0.05 (− 0.15 to 0.05)	0.35
Maximum reward	0.00001 (0.00 to 0.00002)	0.04
Gaming duration	0.37 (− 0.06 to 0.80)	0.09

CI, confidence interval.

### Publication bias

**Figure 6:**
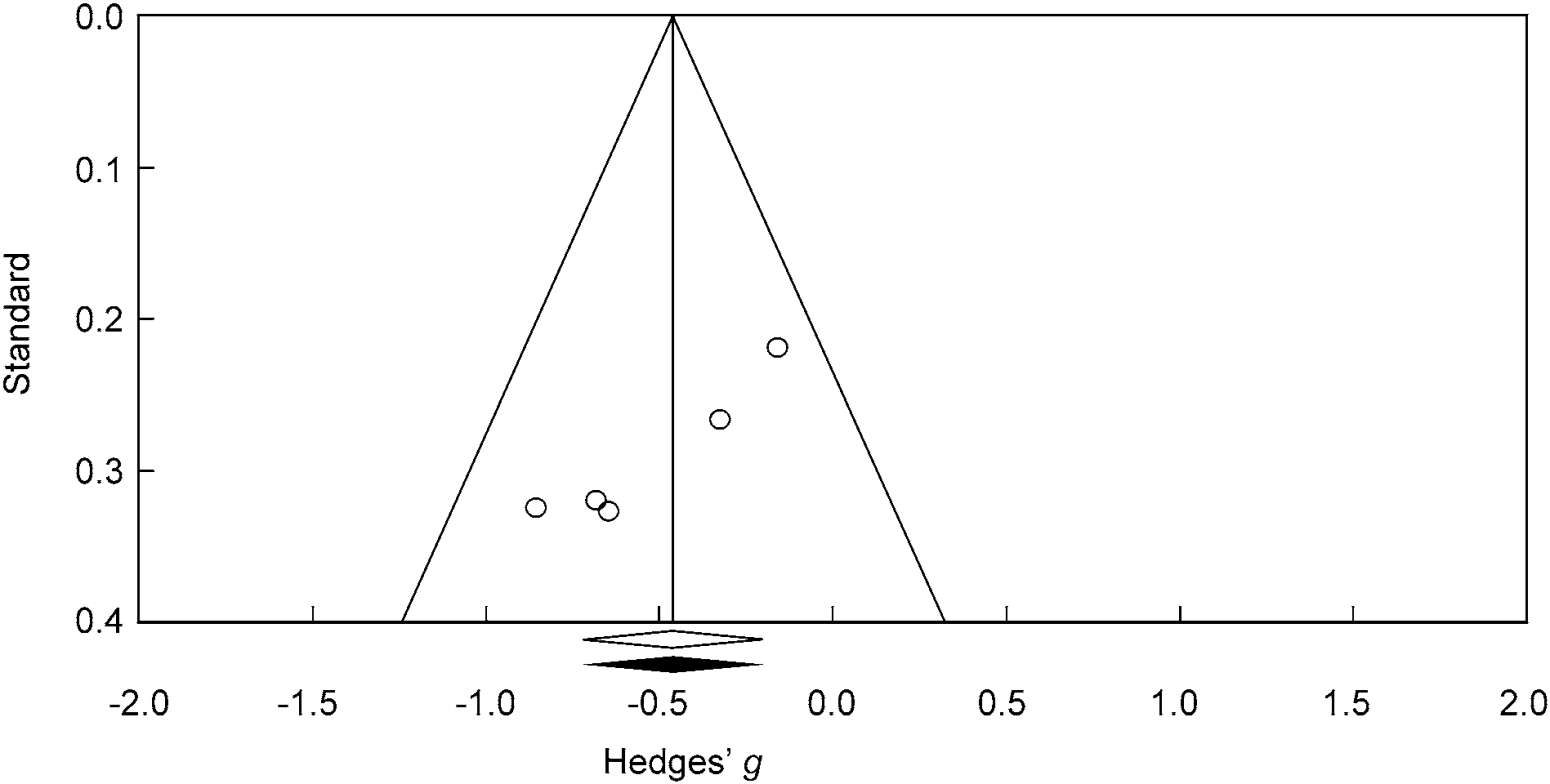
Random-effects funnel plot detailing publication bias in the studies reporting probability discounting between subjects with Internet gaming disorder and healthy controls.

Under a fixed- or random-effects model, visual inspection of the funnel plot revealed significant asymmetry. Because no potentially missing study was imputed on both sides of the plot, we were unable to use the ‘trim and fill’ method to adjust our results (Fig. 6). Besides, Egger’s test was significant (*p* < 0.01). The results, therefore, suggested significant publication bias.

## Discussion

### Main findings

To our best knowledge, the present study is the first meta-analysis to investigate the relationship between IGD and PD. Our results showed that individuals with IGD had a lower degree of PD (i.e., higher subjective values) compared with that in their comparators with moderate effect size (*g* = − 0.46; 95% CI = − 0.72 to − 0.20), suggesting that individuals with IGD tend to overestimate the possibility of a substantial gain and underestimate the likelihood of gaining nothing than their healthy counterpart. This is a phenomenon frequently observed in those with other forms of addiction^41,42^. Moreover, our results further showed that individuals with IGD exhibited a shorter RT than that in healthy controls (*g* = − 0.51; 95%CI = − 0.87 to − 0.15), indicating that individuals with IGD spent less time making risky choices. Taken together, our findings demonstrated that individuals with IGD tend to over-estimate potential gain from playing online games on which they spend a significant proportion of their time, resulting in negligence of social and academic activities that contributes to social or occupational dysfunctions.

Another interesting finding of our study was that, compared with their healthy counterparts, individuals with IGD were more willing to take risks regarding probabilistic gains. However, their performance on probabilistic losses was similar. The findings indicated that individuals with IGD tended to take risk when they expected gains but their risk-averse behavior was similar to that in healthy controls when subject to losses. Our meta-regression further showed that maximum magnitude of reward positively correlated with the PD rate (*p* < 0.04), suggesting that risk-averse behaviors increased with the amount of reward.

Finally, we performed subgroup analysis using two methods, namely, AUC method and *h* value. While we found significant difference in PD between the IGD group and healthy controls by using *h* value, the difference between the two groups was not significant with the AUC approach. The findings suggested that the significance of difference may depend on the analytical methods.

### Significant association between online gaming disorder and risk-taking behaviors

With respect to the nature of IGD, the DSM-V has included the disorder under Section III “Conditions for further study”^1^ and the World Health Organization (WHO) has identified it as an official diagnostic entity in the latest (eleventh) revision of the International Classification of Diseases (ICD-11)^2^. In fact, IGD shared a lot of common features with addictive disorder, especially in the aspects of impulse control and risk taking behaviors^39^. Some studies even suggested that individuals with IGD may have impaired cognitive control in risk assessment and found that their neural connections may be different from those in healthy subjects^43,44^.

The current study may shed light on one of the behavioral mechanisms underlying IGD, namely the discounting of probabilistic gains and losses^45^. Despite previous inconsistent findings about the differences in the rate of PD between subjects with IGD and normal controls, our study showed that the former had a lower PD rate than that in the latter with a moderate effect size, suggesting that subjects with IGD tended to underestimate the consequences of risky behaviors compared to the tendency in healthy controls. Previous studies found that PD rate is associated with risk taking and gamblers who had elevated risk propensity also had lower PD rates than those in controls^16,46^. Therefore, it is possible that the IGD group had impaired prospective thinking which involved risk evaluation to render them vulnerable to easy addiction to online games without considering the potential negative consequences. The finding is supported by that of previous studies investigating the underlying neural mechanism among individuals with IGD, which demonstrated impaired functional connectivity in brain regions involving the reward circuits^24^, less engagement in the executive control network^29^ and less neural response in certain brain regions related to addictive behaviors such as parahippocampal gyrus, the anterior cingulate cortex, and the medial frontal gyrus^39^ compared to those in normal subjects.

Our subgroup analysis further showed that individuals with IGD were more willing to take risks in probabilistic gains compared with their healthy counterparts (Hedges’ g = − 0.42) but there were no difference in their performance on probabilistic losses. The findings may imply that individuals with IGD tended to take risks when they expected gains but their risk evaluation behavior was similar to that in healthy controls on encountering potential losses. This is supported by previous evidence showing an asymmetrical pattern of brain activation on discounting between future gains and future losses^47^. Medial orbitofrontal cortex (mOPFC), mPFC, PCC, and ventral striatum are activated by expected gains, while ACC, insula, superior frontal gyrus (SFG), mPFC and PCC are associated with future losses^47^. Additionally, the activation has been found to be larger in mPFC while participants faced losses^47^. In addition to the tendency to take risks, our analysis further showed that individuals with IGD spent less time making risky choices (*g* = − 0.51) with a moderate effect size. Overall, our results indicated that subjects with IGD tended to make more risky choices and spent less time on the decision-making process compared to those in healthy controls.

### Clinical implications for preventing risk behaviors

The findings of the current investigation had some therapeutic implications and may provide a new target for psychotherapy in patients with IGD by top-down and bottom-up strategies. Top-down strategies, such as working memory training (WMT) and goal management training (GMT), can increase future-based discounting function by rewiring of the dorsolateral and ventrolateral prefrontal cortices^48^. Interventions improving bottom-up impulsive system, such as response inhibition training (RIT), can reduce stimulus-action biases by rewiring of the inferior frontal gyrus (i.e., impulsive biases)^48^. Although the results were only from five studies, our findings suggest that WMT, GMT, and RIT may be employed for the treatment of Internet gaming-related addictive disorders.

Our subgroup analysis further found that participants with IGD suffered from impairment in decision making, mainly involving probabilistic gain rather than loss. To combine our behavioral results with previous evidence^47^, we inferred that neural targets for treating individuals with IGD may be mOPFC rather than mPFC. In other words, enhancing response inhibition, such as RIT, may be a treatment priority. Nevertheless, the result was derived from only two studies. Indeed, contrary to our finding, some previous investigations reported no difference in attitudes toward probabilistic gains and losses^45,49^. Further studies are needed to clarify the difference in risk-taking behaviors between probabilistic gains and losses in individuals with IGD.

### The negative correlation between the magnitude of reward and risk-seeking behaviors

Although our overall results showed a significantly lower PD rate in individuals with IGD than that in healthy subjects, inconsistent results were reported in individual studies possibly due to different methodologies for PD measurement. To be specific, despite the demonstration of an unanimously lower PD rate in all five studies compared to that in normal controls, three showed statistical significance^24,29,39^ but the other two did not^37,38^. Besides, when we take the maximum magnitude of reward into consideration, both studies using higher magnitude of reward failed to show difference in PD rate between the IGD and control groups^37,38^. In fact, according to the prospect theory, people tend to take higher risks with smaller amount of money^50^. Interestingly, our meta-regression also showed a positive correlation between the maximum magnitude of reward and the PD rate (*p* < 0.04), suggesting a negative association between individuals’ risk-seeking behaviors and the amount of rewards (i.e., tendency of taking a lower risk with a larger amount of reward). Therefore, it is possible that the experimental settings in the two studies with a larger amount of reward^37,38^ may not be sensitive enough to detect the difference in risk-seeking behaviors between the IGD and healthy control groups. Moreover, participants in those two studies^37,38^ were younger than those in the other three^24,29,39^ and one study only included adolescent rather than adults^38^. It is rational to speculate that, compared with older individuals, younger people may have a weaker financial basis to support their taking a high risk for a large amount of potential gains. On the other hand, our meta-regression failed to demonstrate a significant impact of age on PD rate. Therefore, despite the preliminary nature of this finding, the present study showed the importance of taking the experimental design especially the amount of reward into consideration for PD investigation in the IGD setting.

### The issue of using different methodologies for measuring risk behaviors

Our subgroup analysis with the *h* value, rather than AUC, revealed a significantly lower PD in the IGD group compared with that in the controls. Nevertheless, taking Hedge’s g into account, there was no significant difference between the two analytic approaches (*p* = 0.06). Moreover, as mentioned above, both studies adopting AUC also used a larger amount of reward, which our meta-regression identified as a potential factor affecting the PD rate. Hence, such a difference in experimental settings among the included studies may be a confounding factor that needs to be addressed. Although there may be a difference between measurements using parameters of a discounting function and AUC^30^ as in delay discounting, in which a study suggested that AUC may be a more sensitive tool^51^, there is no previous evidence showing how this difference may influence study outcomes regarding PD. Therefore, further investigations are warranted to support our preliminary findings regarding the impact of using different analytic methods (i.e., *h* value and AUC) in the setting of probability discounting.

### Limitations

The present study had its limitations. First, only five studies were included with the involvement of a total of merely 300 individuals; therefore, our results were only preliminary and needed more supports from further large-scale clinical studies. Nevertheless, because of scant research in this field and also limited information on the behavioral patterns of IGD, our study may open an important avenue for further research. Second, because all studies were from China, the findings of the present meta-analysis may not be extrapolated to individuals of other ethnical and cultural backgrounds who have been shown to exhibit different severities of internet gaming distress because of variations in culture-specific achievement motivations, social connection, and psychosomatic experiences^52^. Indeed, although Internet gaming disorder may be more prevalent in Asian countries, it has already become a global issue^53^. Our study could serve as a reference for further studies focusing on possible ethnical differences in this disorder. Third, there was some evidence of publication bias as visual inspection of the funnel plot revealed significant asymmetry. This may be explained by different experimental settings of two groups in which two studies in one group used YQD, AUC and a higher amount of maximal rewards^37,38^, while three studies in the other group used YIAT, *h* values and a smaller amount of maximal rewards^24,29,39^. However, because there were only five studies included in our meta-analysis, more studies with similar designs may be required to support our finding. Fourth, the diagnosis of IGD was not standardized in the included studies, two of which used YQD for IGD diagnosis and shared a very similar experimental setting including the use of AUC and a larger amount of reward. Therefore, it was difficult to differentiate the potential influences of different factors on the final results. Notwithstanding the above limitations, our findings suggest that different experimental settings and diagnostic tools may have potential effects on study results, underlying the importance of taking these factors into consideration during research design. Fifth, our keyword focused mainly on internet addiction and did not include keywords focusing on gaming addiction; therefore studies regarding this topic may be overlooked in this meta-analysis. Finally, as an inherent shortcoming of a meta-analysis, we could only evaluate the significance of an association rather than establish a causal relationship. Nevertheless, our findings provide a direction for further study to focus on specific neural function regarding probabilistic discount in IGD individuals.

## Conclusions

The results of the present study showed that individuals with IGD spent less time making impulsive decisions and tended to overestimate the potential gains associated with the consequences of risk-based decisions, especially when the potential gains were not substantial. Compared with healthy individuals, those with IGD were more willing to take risks in probabilistic gains despite the lack of difference regarding probabilistic losses between the former and the latter. Our findings not only supported the use of probability discounting for assessing individuals with IGD but may also provide new insights into appropriate interventions.

## Data Availability

The data generated during the analysis are available on reasonable request made to the corresponding authors.
